# Curcumin Release from Biomaterials for Enhanced Tissue Regeneration Following Injury or Disease

**DOI:** 10.3390/bioengineering10020262

**Published:** 2023-02-16

**Authors:** Adelle E. Hamilton, Ryan J. Gilbert

**Affiliations:** 1 Department of Biomedical Engineering, Rensselaer Polytechnic Institute, 110 8th Street, Troy, NY 12180, USA; 2 Center for Biotechnology and Interdisciplinary Studies, Rensselaer Polytechnic Institute, 110 8th Street, Troy, NY 12180, USA; 3 Albany Stratton Veterans Affairs Medical Center, 113 Holland Avenue, Albany, NY 12208, USA

**Keywords:** curcumin, biomaterials, electrospun fibers, nanoparticles, hydrogels, scaffolds, regeneration, drug delivery

## Abstract

Curcumin, a bioactive phenol derived from turmeric, is an antioxidant, anti-inflammatory, and antibacterial molecule. Although curcumin exhibits beneficial effects in its innate form, it is highly hydrophobic, which leads to poor water solubility and, consequently, low bioavailability. The lack of bioavailability limits curcumin’s effectiveness as a treatment and restricts its use in clinical applications. Furthermore, to achieve beneficial, clinically relevant results, high doses of curcumin are required for systemic administration. Many researchers have utilized biomaterial carriers, including electrospun fibers, nanoparticles, hydrogels, and composite scaffolds, to overcome curcumin’s principle therapeutic limitation of low bioavailability. By using biomaterials to deliver curcumin directly to injury sites, researchers have harnessed the beneficial natural properties of curcumin while providing scaffolding to support tissue regeneration. This review will provide an in-depth overview of the literature that utilizes biomaterial delivery of curcumin for tissue regeneration in injury and disease models.

## 1. Introduction

Turmeric, a bright yellow spice, is a commonly used food coloring or additive. However, most of its history lies in its prolific use in a variety of traditional medicines throughout southeast Asia. Turmeric has been documented as a treatment for respiratory conditions, diseases associated with abdominal pain, rheumatism, diabetic wounds, and inflammation [1,2,3]. The anti-inflammatory and antioxidant properties of turmeric are due to its major bioactive compound: curcumin. In recent decades, researchers have investigated curcumin’s beneficial effects and its role in diseases and disorders. Confirming and adding to its uses in traditional medicine, curcumin exhibits anti-inflammatory, antioxidant, tissue protective, chemoprotective, antibacterial, antiviral, and immunomodulatory roles [3,4,5,6,7,8,9,10,11,12,13,14,15,16,17,18,19,20,21]. Although the benefits of curcumin in medicine are apparent, curcumin’s effectiveness is diminished due to its hydrophobic properties and rapid metabolism when applied in its base form [5]. Therefore, the ability to deliver curcumin via biomaterials locally to an injury site or for disease applications is of increasing interest.

Biomaterials play a critical role in tissue engineering (TE) as they provide the support and scaffolding necessary for cellular migration and tissue regeneration [22]. Scaffolds can be decorated with or fabricated from extracellular matrix (ECM) molecules, an intricate bioactive network of proteins and glycomolecules unique to each tissue type that contributes to the mechanical and chemical properties of tissues. Additionally, the ECM consists of structural proteins and is a reservoir for growth factors and signaling molecules, which regulate cellular functions such as proliferation and apoptosis [23,24,25]. Biomaterials, both natural and synthetic, are designed to mimic the ECM to promote proper tissue regeneration. However, the biomaterial used is not universal, and the type selected should be specific for the application type. For example, electrospun fibers are widely studied in skin, bone, and neural regeneration applications, while hydrogels are researched for organ, cartilage, and skin regeneration applications [26,27,28,29,30,31].

In addition to providing the necessary support for tissue regeneration, biomaterials may also be designed as drug-delivery vehicles. Many biomaterials can be tailored to release therapeutics for extended periods of time within the body, usually locally at the site of interest. This local delivery of a drug, as opposed to a systemic delivery method, reduces toxicity and limits off-target side effects as a smaller amount of drug is needed to achieve the desired therapeutic effect [32]. With many hoping to harness curcumin’s beneficial effects, current research has focused on curcumin drug delivery from biomaterials. To date, many review articles have been written regarding curcumin; however, these reviews have focused on either a single biomaterial strategy or therapeutic application [33,34,35,36,37,38,39]. This review provides an overview of curcumin, its therapeutic mechanisms and shortcomings in its natural form, and strategies currently used to deliver curcumin from various biomaterial platforms for tissue regeneration following injury or disease.

## 2. Curcumin

**Figure 1:**
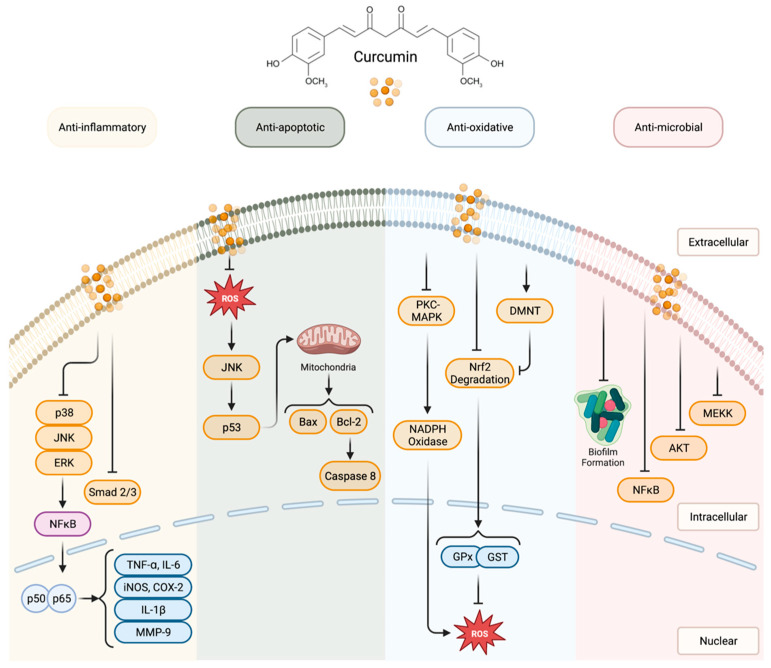
Schematic of the biological mechanisms of curcumin, including anti-inflammatory, anti-apoptotic, anti-oxidative, and anti-microbial pathways. Created with Biorender.com.

Curcumin, chemically known as 1,7-Bis(4-hydroxy-3-methoxyphenyl)hepta-1,6-diene-3,5-dione, is a naturally occurring compound found in plants of the Curcuma longa species. Found in turmeric, curcumin is widely used throughout the world. For example, it is commonly used as a spice for meals such as curry or served in drinks as a flavoring. Turmeric is also used as a coloring agent in cosmetics and fabric dyes. Furthermore, turmeric is used in traditional medicines throughout East Asia [4]. Curcumin is the principal and most widely studied curcuminoid of turmeric and exhibits many health-promoting effects [40]. Biologically, curcumin has diverse effects, providing health benefits to many different tissue types to act in tissue protective, chemoprotective, immunomodulatory, and antibacterial roles (Figure 1). Many of these effects are attributed to curcumin’s anti-inflammatory and antioxidant properties [7]. However, one major limitation in using curcumin as a therapeutic is its limited bioavailability, even when administered at high dosages.

**Figure 2:**
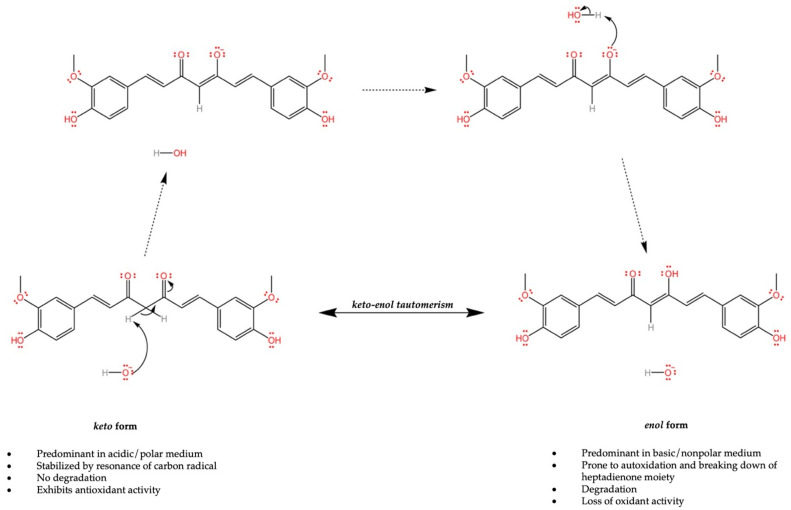
Schematic of the keto-enol tautomerism that takes place within curcumin with intermediate steps shown. Each form is predominant under specific conditions and possesses different properties.

Curcumin consists of three major functional groups: an aromatic O-methoxy phenolic group, an alpha, beta-unsaturated beta-diketone moiety, and a seven-carbon linker [41]. However, it is the two aromatic phenolic groups and diketone moiety that enable the bioactivity of curcumin. Curcumin naturally resides in the enol form since this form is the most energetically stable in the solid phase; however, it also undergoes degradation in basic and non-polar mediums [42,43]. The keto form, preferred for its antioxidative properties, exists in acidic and polar mediums, and undergoes spontaneous keto-enol tautomerism at a physiological pH, converting curcumin into the readily degradable enol form (Figure 2) [42]. Curcumin acts as an antioxidant in its keto form by donating a hydrogen atom from the methylene group (-CH_2_), where the resulting carbon radical is stabilized by resonance [44]. Furthermore, the enol form, which has higher pro-oxidant activity, reacts mainly by electron transfer. The tautomerism of the heptadienone moiety contributes greatly to the antioxidant ability of curcumin, as either the enol or keto forms of curcumin are predominant at either a basic or acidic pH. During the degradation of curcumin at a basic pH, the heptadienone moiety is broken up, leading to the disappearance of the active methylene group and the loss of its antioxidant properties [45].

Curcumin’s anti-cancer properties are dependent on the presence of hydroxyl (-OH) groups in the phenolic ring. Additionally, the methoxy (-O-CH_3_) group increases its antioxidant properties, with the antioxidant properties decreasing with fewer methoxy groups [46]. However, degradation in the enol form is caused by autooxidation, resulting in free-radical formation [47]. Interestingly, when taken in its base form, curcumin exhibits low toxicity. Systematic trials and preclinical studies in animals, including rats, mice, dogs, and monkeys, demonstrate good tolerability of doses between 4000 and 8000 mg/day, with some even demonstrating the tolerability of curcumin at a high dose of 12,000 mg/day [48]. Additionally, pilot and phase I clinical studies for malignant conditions and colorectal cancer demonstrate the tolerability of curcumin doses up to 8000 mg/day, with many studies noting that there is a low systemic bioavailability following oral dosing due to low absorption by the small intestine and enterocyte binding to curcumin [49,50,51].

Even though high doses are applied clinically, curcumin’s efficacy is limited due to its poor stability and low solubility. Many studies concur that the solubility of curcumin is below 8 µg/mL, with several citing ranges between 0.6 µg/mL and 11 ng/mL [52,53,54,55]. Lao et al. demonstrated that when delivered orally at a high dose of 10–12 g/mL in humans, curcumin levels in serum remained at approximately 50 ng/mL [56]. Thus, although highly soluble in organic solvents such as dimethyl sulfoxide (DMSO), acetic acid, and acetone, it is widely agreed that curcumin is insoluble in water [57]. When considering utilizing a therapeutic with low solubility for drug delivery applications, appropriate analytical quantification methods must be used in order to detect its presence. Some of the most commonly used detection methods to quantify curcumin are high-performance liquid chromatography (HPLC), ultra-high-performance liquid chromatography (UHPLC), ultraviolet-visible spectrophotometry (UV-Vis), and fluorimetry, each with their own advantages and limitations. Additionally, due to its low solubility and poor stability, the selection of release medium plays a role in quantification. Many studies presented in this paper opted to use either phosphate-buffered saline (PBS), in which curcumin has a higher solubility than in water, or a mixture of ethanol and water. While the ratios of ethanol to water vary, one frequently used combination is 50% ethanol:50% water; Nasra et al. demonstrated that 50% ethanol in the release medium was critical in maintaining curcumin stability [58]. An overview of quantification methods, their advantages and limitations, and release mediums is provided in Table 1. Thus, in order to increase overall stability and solubility of curcumin, many researchers have begun developing biomaterials for local curcumin delivery.

## 3. Curcumin Delivery from Electrospun Fibers

Electrospinning is a technique that creates micro- or nanometer-sized polymeric fibers by applying a high voltage to a needle in which the polymer solution resides. There are four major components necessary to create electrospun fibers: a high-voltage power supply, a syringe pump, a reservoir (typically a needle), and a conductive collector [79]. When a high voltage is applied to the reservoir, electrification occurs, leading to a charged jet release of polymer. As the polymer whips through the air and stretches into thinner diameters, the polymer solidifies and is collected on a grounded collector [80]. Depending on the collector used, electrospun fibers can be either aligned or collected as an unorganized fibrous mat, where fiber orientation dictates their subsequent use in tissue engineering and regeneration. As fibrous mats, electrospun fibers may be used as wound dressings or bone scaffolds. For wound healing applications, the orientation of electrospun fibers is similar to the orientation of the ECM in the skin, which enables cell adhesion, growth, and proliferation [81]. Furthermore, electrospun fiber mats offer a physical protective barrier due to the small gaps, specifically small porous features, between the fibers, which also allow for oxygen permeability and fluid drainage [82,83]. Additionally, the highly porous structure of the fibrous mats, which is similar to the ECM of bone, promotes the cellular differentiation of osteoblast-like cells (SaOS-2 cells) into osteocytes [84,85].

### 3.1. Skin Tissue Engineering

#### 3.1.1. Full-Thickness Wound Healing Models

Curcumin delivery from electrospun fibers has demonstrated great promise for wound healing applications, with many researchers adopting the blending electrospinning technique to incorporate curcumin within the polymer fiber. Additionally, full-thickness wound models are often employed to investigate a drug or biomaterial’s effect on wound contraction, angiogenesis, and closure [86]. Fu et al. reported that curcumin-loaded poly(ε-caprolactone)-poly(ethylene glycol)-poly(ε-caprolactone) (PCEC) fibrous mats demonstrated a wound closure ratio of 93.3 ± 5.6% 21 days after application to a full-thickness wound model in adult female Wistar rats [60]. With a curcumin release rate of 66.4% after ten days quantified using high-performance liquid chromatography (HPLC), the 10%-Cur/PCEC treatment group outperformed the PCEC (80.4 ± 93%) and control (no fiber) (76.9 ± 4.9%) groups. Additionally, mice treated with the 10%-Cur/PCEC scaffold exhibited faster re-epithelialization and blood vessel formation, as well as an increase in collagen deposition over time, visualized by hematoxylin and eosin (H&E) and Masson’s trichrome (MT) staining.

#### 3.1.2. Diabetic Wounds

Ranjbar-Mohammadi et al. demonstrated that curcumin-loaded poly(ε-caprolactone)/gum tragacanth (PCL/GT/Cur) electrospun fibers increased collagen content and accelerated the wound healing process in diabetic male Sprague-Dawley rats [65]. Following the fabrication of their electrospun fibers, curcumin release was analyzed utilizing ultraviolet-visible spectroscopy (UV-Vis) over 20 days, where PCL/GT/3%-Cur exhibited a release rate of 65% [87]. Fifteen days post-application of PCL/GT/Cur fibers into full-thickness wounds, the wounds had closed entirely, while the wound area of the controls that received no scaffold decreased to 20.96 ± 1.35%. Additionally, wounds that received fibers containing curcumin demonstrated better collagenous regeneration and faster re-epithelialization in the early stages of healing compared to the controls, as visualized with H&E and MT stains. The researchers also sought to determine the antibacterial properties of their PCL/GT/Cur fibers and determined that their nanofibers were 99.9% and 85.14% effective at eliminating methicillin-resistant Staphylococcus aureus (MRSA) and extended-spectrum β-lactamases (ESBL), respectively.

An increased rate of wound closure was also observed in a diabetic mouse model following curcumin delivery via PCL fibers [88]. In this study, published by Merrell et al., two different concentrations of curcumin were used: 3% and 17% (*w*/*w*), where the 3%-curcumin-loaded PCL fibers and the 17%-curcumin-loaded PCL fibers had released ~20 µg and ~35 µg of curcumin after three days, respectively, quantified via fluorimetry. Following liposaccharide (LPS) stimulation of macrophages, the researchers observed that both the 3%-curcumin-loaded and 17%-curcumin-loaded PCL fibers modulated the anti-inflammatory activity of macrophages in vitro, depicted by a decrease in IL-6 release. The decrease in IL-6 was observed to be concentration-dependent, where the 17%-loaded fibers exhibited significantly lower IL-6 release from macrophages than the 3%-loaded fibers. Additionally, both concentrations of curcumin-loaded fibers exhibited cytoprotective effects when HFF-1 fibroblasts were exposed to hydrogen peroxide insults. Interestingly, in vitro cell viability assays revealed lower cellular viability when cultured on either PCL/curcumin groups compared to the control PCL fibers. However, when analyzed in vivo using a dermal punch wound, mice treated with PCL/curcumin-17% fibers demonstrated ~80% wound closure compared to the ~60% wound closure when treated in the control PCL nanofiber group after 10 days.

### 3.2. Bone Tissue Engineering

Curcumin also demonstrates promise for bone tissue regeneration applications, as tested in an adult mongrel dog alveolar bone defect model. To perform this study, Ghavimi and colleagues utilized a composite biomaterial construct. Traditionally, composites are composed of two or more materials to create a final product with enhanced material properties compared to the two individual materials [89]. Due to their novel properties, including increased biological properties and tunable drug release rates, they have advanced the field of drug delivery [90]. Within this literature review, composites will consist of the combination of two or more different drug delivery systems, including electrospun fibers, nanoparticles, and hydrogels.

Ghavimi et al. performed a study utilizing an asymmetric membrane, with a collagen nanofiber and poly(lactic-co-glycolic acid) (PLGA)-aspirin nanoparticle base on one side (PACNFs) and curcumin and collagen nanofibers on the other side (CCNFs) [91]. To determine the antibacterial effects, the membrane was evaluated against *S. aureus*, *E. faecalis*, and *E. coli* bacteria. Although no curcumin release rate was reported for the CCNF side, results from the bacteria test demonstrated this side could reduce bacterial counts of *S. aureus*, *E. faecalis*, and *E. coli*, with the greatest effectiveness against *S. aureus*. In contrast, the PACNF side did not exhibit antibacterial effects. When examined for gene expression in vitro using Western blotting, both the CCNF and PACNF membranes showed increased RNA and protein expression of the Runx-9 and osteocalcin (OCN) osteogenic genes in dental pulp stem cells (DPSCs), as well as increased proliferation of DPSCs. Twenty-eight days after the induction of a bone defect, H&E images depicted new bone formation, suggesting that the PACNF later facilitated osteoinduction and the CCNF layer promoted osteoconduction, improving on the actions of the PACNF layer, while the control defects covered with a commercial membrane exhibited no bone regeneration. Curcumin delivery from electrospun fiber biomaterials is summarized in Table 2.

## 4. Curcumin Delivery from Nanoparticles

Electrospun fibers function as both a drug depot and a scaffold, providing the necessary support to regenerating tissues. However, in cases where scaffolding may not be necessary, nanoparticles serve as highly efficient drug-delivering molecules, often improving the bioavailability of the loaded compound of interest [92,93]. The fabrication of nanoparticles (NPs) utilizes one of five different methods: solvent evaporation, emulsification/solvent diffusion, nanoprecipitation, ionic gelation, or emulsification/reverse salting-out [94]. Polymeric nanoparticles are widely used for drug-delivery applications due to their small size, ranging from between 1 and 100 nanometers (nm) [95]. Their size increases bioavailability and allows for curcumin delivery to cells and tissues, in addition to their usual advantages of being protective of their drug cargo and their potential for controlled drug release [96].

### 4.1. Skin Tissue Engineering

#### 4.1.1. Full-Thickness Wound Healing Models

Many researchers have investigated the delivery of curcumin from nanoparticles for wound healing applications. With the use of nanoparticles, it is important to note the difference between drug loading efficiency and encapsulation efficiency. The former refers to the amount of drug loaded per unit of weight in the nanoparticle, while the latter refers to the amount of drug entrapped in the nanoparticle. Chereddy et al. synthesized PLGA-curcumin nanoparticles (PLGA-CC NPs) that increased wound healing activity compared to PLGA or curcumin alone in a full-thickness excisional mouse wound model [61]. Following the synthesis of the NPs utilizing the oil/water emulsion-solvent evaporation technique, researchers reported a curcumin encapsulation efficiency of 89.2 ± 2.5%, with a release rate of curcumin at 75.7 ± 3.4% after 8 days. The model used in this study was a RjHan:NMRI mouse, an Albino outbred strain commonly used for pharmacology and toxicology, with full-thickness excisional wounds created using an 8 mm round skin biopsy punch. When injected intradermally at a dose of 1 mg into splinted female RjHan:NMRI mice, the PLGA-CC NP treatment group demonstrated two-fold enhanced accelerated healing compared to the curcumin-only and nanoparticle-only controls. Unlike the control groups, the curcumin nanoparticle group enabled complete re-epithelialization and significantly higher production of collagen after 10 days, as observed by H&E staining. Furthermore, the PLGA-CC NP treatment reduced inflammatory infiltration activity and inhibited the expression of inflammatory mediators, demonstrated by a significant decrease in myeloperoxidase (MPO) activity, as well as a significant downregulation in mRNA expression of the antioxidative enzyme glutathione peroxidase (GPx) and inflammatory transcription factor, NFκB. GPx triggers the generation of reactive oxygen species (ROS) at wound sites which, in turn, activate the NFκB pathway [97,98]. This demonstration of ROS quenching could potentially be attributed to curcumin’s antioxidative properties; the curcumin in the PLGA-CC NPs dampened ROS, leading to the decrease in GPx expression, reducing NFκB activation and its resulting expression.

In a study by Zahiri et al., improved wound closure was also observed when using an electrospun PCL and gelatin (Gela) scaffold containing curcumin-loaded chitosan nanoparticles (PCL/Gela/NCs/Cur) [66]. Fabrication of the curcumin-loaded chitosan nanoparticles (NCs) (NCs/Cur) was accomplished through ionic gelation. Through characterization of the curcumin-loaded NCs and ultraviolet-visible spectrometry, researchers reported 4.2 ± 0.2% drug loading, encapsulation efficiency of 93 ± 5%, and complete release (100%) achieved and ~83% release achieved from NCs/Cur after four days and PCL/Gela/NCs/Cur after ten days, respectively. Following topical application to a full-thickness wound model in male Wistar rats, the PCL/Gela/NCs/Cur and NCs/Cur condition groups significantly increased wound closure percentage compared to the PCL, PCL/Gela, and negative control groups. Interestingly, although there was a statistical difference in wound closure percentage on day seven between the PCL/Gela/NCs/Cur group and NCs/Cur, no statistical difference was present on day 14. Finally, H&E and Masson’s trichrome (MT) staining images of wounds treated with the curcumin-releasing materials demonstrated better epidermal proliferation and increased collagen synthesis and angiogenesis when compared to the control groups.

#### 4.1.2. General Wound Healing Models

Manca et al. developed curcumin-immobilized vesicles, termed hyalurosomes, for inflammation and wound healing applications in female CD-1 mice, which are an Albino outbred multipurpose strain commonly used in toxicology and pharmacology research [99]. The initial encapsulation efficiency of curcumin was determined to be ~79% for hyalurosomes. Following an in vitro scratch assay utilizing human keratinocytes and a topical application of the curcumin-loaded hyalurosomes, the free curcumin dispersion group showed partial wound healing, while the curcumin-loaded vesicle treatment group allowed for complete scratch closure after 48 h. Additionally, using an in vivo skin damage assay, the curcumin-hyalurosome group avoided damage and the loss of superficial skin following daily 12-O-tetradecanoylphorbol-13-acetate (TPA) application, visualized by the measurement of MPO, when compared to free curcumin and curcumin-liposome control groups after six days. This better performance by the curcumin–hyalurosome group is likely due to the combined effect of curcumin and hyaluronan, a glycosaminoglycan known for its beneficial role in wound healing [100,101].

Bajpai et al. examined the regenerative properties of their curcumin construct, in which cellulose nanocrystals and Curcumin/Silver (Ag) nanoparticles were loaded into chitosan films [102]. This construct was tested using an in vivo skin irritation model on Albino Wistar rats, where the curcumin-containing chitosan approach passed the test as it did not induce irritation. Following an in vivo wound healing study, the treatment group (Ch/CNC (Ag Np/Cur)) enabled a wound reduction of 98%, compared to a wound reduction of 57% in the curcumin-only patch group and a wound reduction of 48% in the no curcumin control group after 25 days. Furthermore, the treatment group demonstrated less scarring at the wound site, as visualized with histological evaluation, which may be due to an increase in angiogenesis and collagen formation.

Lastly, curcumin nanoparticles (Cur NPs) have been incorporated into polyvinyl alcohol (PVA)/collagen composite films (CPCF) to promote wound healing in a male Sprague-Dawley model [62]. Using high-pressure liquid chromatography (HPLC), Leng et al. demonstrated a drug loading and encapsulation efficiency of 9.61 ± 0.12% and 96.09 ± 1.21%, respectively. Additionally, after five days, Leng and colleagues observed a 90% curcumin release from the Cur NPs and a 76% curcumin release from their CPCF construct using HPLC. When analyzing in vitro cytotoxicity with human skin fibroblasts, Cur/PCEC NPs reduced the toxic effects of curcumin commonly observed with non-biomaterial delivery. Additionally, in vivo applications demonstrated that full-thickness wounds in the CPCF group healed faster than those of other groups over 15 days, with the treatment group revealing an average wound healing rate of ~92%, while the untreated control group showed a slower rate of ~75% on day 9. However, by day 15, all constructs displayed similar healing rates, with a 98.03 ± 0.79% rate in the CPCF group outperforming the untreated control group, which had an average wound healing rate of 92.73 ± 1.83%. Furthermore, the treatment group had an increase in collagen and vascularization, along with decreasing the inflammatory response, as visualized with H&E and MT staining.

### 4.2. Musculoskeletal Engineering

#### Tendon Rupture and Repair Model

In addition to wound healing, curcumin release from nanomaterials is beneficial to tendon and muscle healing. Usually seen as a complication of surgical intervention following tendon ruptures, tendon adhesion is triggered by the healing process when surrounding granulation tissue and tenocytes infiltrate the injury site, resulting in the accumulation of fibrotic tissue [103,104,105]. Thus, adhesion formation is thought to be one of the most critical factors that impact functional restoration following injury, as they are detrimental to the mechanical properties of the tendon [103,106]. To analyze the effect of curcumin on tendon adhesion and healing, Zhang et al. utilized curcumin-loaded nano-micelles (gold nanorods [GNRs]-1/curcumin in polymeric micelles [curc@PMs]) (GNRs-1/curc@PMs) that had an encapsulation efficiency of 41%, determined via UV-vis [107]. Sprague-Dawley rats underwent tendon rupture and repair (TRR) procedures, and the experimental solution was injected into the surgical site at a dosage of 0.44 mg curcumin/kg in 0.1 mL saline. After ten days, all TRR wounds had healed, but there was significantly less adhesion in the experimental GNRs-1/curc@PMs group compared to the controls: curcumin dissolved in saline and saline only. Additionally, using mechanical testing, the tensile strength and toughness of the Achilles tendons were tested, where the treatment group exhibited significantly more range of motion. Furthermore, H&E staining demonstrated there was less inflammation and tendon adhesions than when the TRR was treated with curcumin dissolved in saline or saline alone.

Kazemi-Darabadi et al. compared the effects of curcumin and curcumin-loaded nanomicelles on Sprague-Dawley rat muscle healing following the partial transection of the gastrocnemius muscle [108]. After surgery, the rats received a daily oral gavage of either saline, free curcumin at 500 mg/kg, or nanocurcumin at 100 mg/kg per dose. To obtain nanocurcumin, this study utilized commercially available curcumin-loaded nanomicelles, which were mixed with normal saline to obtain a 15 mg/mL concentration of nanocurcumin. Histologically, visualized using H&E and MT stains, there was a significantly lower percentage of collagen fibers, muscle fiber regeneration was significantly higher, and blood vessel formation was significantly more when the treatment included the curcumin nanoparticles compared to sham and saline-only control groups after two weeks. Therefore, encapsulating curcumin into nanoparticles increases its bioavailability and enhances its effects in the muscle injury model.

Lastly, Mahdy et al. investigated the effects of chitosan (Cs-NPs) and curcumin nanoparticles (Cn-NPs) on fibrosis and regeneration following a glycerol-induced muscular injury in Wistar rats [109]. Fabrication of the curcumin-poly(ε-caprolactone) nanoparticles occurred using the single emulsion-solvent evaporation technique. Four types of NPs were produced with differing curcumin concentrations: Cn-5, Cn-15, Cn-20, and Cn-40, corresponding to a dose of 5, 15, 20, and 40 µg of curcumin in the Cn-NPs. Following the induction of injury, rats received a first intraperitoneal injection of Cn-NPs in 1 mL PBS one hour after injury, and a second dose on day four after muscle injury. At the end of the study, seven days after glycerol injury induction, histological images revealed fibrosis was decreased in a dose-dependent manner in the Cn-NPs group, with a decrease of 28%, 48%, and 60% in the 5, 20, and 40 µg groups compared to the glycerol-only controls. Furthermore, there was reduced inflammation, as observed by the decreased number of CD-68+ cells in the 20 and 40 µg groups by 67% and 76%, respectively, as well as decreased collagen deposition, seen by the decrease in Col-1+ area by 49% and 61%, respectively.

### 4.3. Nervous System Engineering

#### 4.3.1. Huntington’s Disease

Due to the highly restrictive nature of the blood–brain barrier and curcumin’s poor bioavailability, curcumin has limited effects on the central nervous system (CNS). However, biomaterial delivery of curcumin may improve the bioavailability of curcumin to the CNS. In a study by Pepe et al., biodegradable curcumin-encapsulated hyaluronic acid-palmitate nanoparticles (Cur-HA-palmitate NPs) reduced neuronal apoptosis in an in vitro Huntington’s disease (HD) model [67]. The Cur-HA-palmitate NPs used in this study were fabricated via emulsification/solvent diffusion and, when evaluated with UV-vis after 72 h, had released ~70% of the encapsulated curcumin. Using a widely used HD model with conditionally immortalized mouse striatal knock-in cells expressing mutant huntingtin, it was observed that the Cur-HA NPs had greater cell permeability, allowing for cellular penetration and subsequent intracellular drug delivery compared to curcumin alone. Additionally, there was a significantly reduced cell susceptibility to apoptosis in the curcumin nanoparticle group, indicating that it has neuroprotective effects in vitro for Huntington’s disease.

Also seeing a potential use for curcumin for Huntington’s disease applications, Sandhir et al. used curcumin-encapsulated solid lipid nanoparticles (C-SLNs) to diminish the effects of 3-nitroproprionic acid (3-NP)-induced HD in rats [68]. After disrupting the SLN dispersion using chloroform and methanol, the total drug content within the particles was estimated spectrophotometrically to be ~93.25 ± 1.85%, with a release of 53.77 ± 2.45% over 6 h. Following the induction of HD to female Wistar rats, the C-SLNs treatment group had a significant restoration of mitochondrial complex activity and cytochrome levels, in addition to a reduction in mitochondrial swelling, lipid peroxidation, and reactive oxygen species when compared to the 3-NP-treated group. Furthermore, in vivo testing showed that C-SLNs successfully increased locomotor activity and reduced gait abnormalities in the 3-NP group. Seven days later, on the narrow beam walk test, the completion time for 3-NP-treated animals was increased by 73%, which was decreased by 37% following the administration of C-SLNs. Furthermore, compared to the saline control animal group, the average speed of the 3-NP group was reduced by 40% and increased by 58% after curcumin administration, indicating that nanocurcumin has the potential to mitigate some impairments caused by Huntington’s disease.

#### 4.3.2. Alzheimer’s Disease

One other neurodegenerative disorder where curcumin may diminish the symptoms is Alzheimer’s disease (AD). Tiwari et al. utilized curcumin-encapsulated PLGA nanoparticles (Cur-PLGA-NPs) to investigate neurogenesis and neuronal differentiation in vivo [69]. Similar to previous studies, the nanoparticles were fabricated using the emulsion–solvent evaporation method. The nanoparticles exhibited a dual release profile, where ~60% of the curcumin was freed initially, followed by a sustained delivery to total ~74% curcumin release over seven days. It was observed that the Cur-PLGA-NPs significantly enhanced neural stem cell (NSC) proliferation in the hippocampus and subventricular zone (SVZ) when compared to the control vehicle, empty PLGA-NP, and bulk-soluble curcumin groups. Neuronal cell counting was performed by bilaterally counting BrdU/NeuN labeled cells in six sections at 600× *g* magnification in the dentate gyrus and SVZ and averaging the number for each rat, and demonstrated an increase in cell differentiation in the Cur-PLGA-NP group compared to bulk curcumin. In an in vivo Aβ-induced Wistar rat model of Alzheimer’s disease, four treatment groups were utilized to determine the effectiveness of the Cur-PLGA-NPs compared to curcumin alone (bulk curcumin, BC): Aβ+BC (0.5 mg/kg), Aβ+Cur-PLGA-NPs (0.5 mg/kg), Aβ+BC (20 mg/kg), and Aβ+Cur-PLGA-NPs (20 mg/kg). Following application, there was a significant increase in BrdU/DCX co-labeled cells in the Cur-PLGA-NP groups, indicating enhanced neuronal differentiation and reduced AD-mediated inhibitory effects on neurogenesis compared to the bulk curcumin groups at the same dosages. Interestingly, Cur-PLGA-NPs at 20 mg/kg allowed for a greater reversal of the cognitive dysfunction associated with AD and inhibited neurodegeneration, while BC (20 mg/kg) and Cur-PLGA-NPs (0.5 mg/kg) had similar effects. From this study, it is demonstrated that curcumin nanoparticles are effective at improving neurogenesis at lower doses, while a much higher dose of bulk curcumin is needed to achieve the same effect.

#### 4.3.3. Stroke Model

Upon restoration of the blood flow following a stroke, cerebral ischemia-reperfusion (CIR) injury occurs, which often causes irreversible neuronal injury and death by generating large amounts of reactive oxygen species. In one study that attempts to reverse the detrimental effects of stroke, Mukherjee et al. fabricated curcumin-containing polyethylene glycol (PEG)-ylated PLGA nanoparticles (NC) using a modified emulsion-diffusion-evaporation method for CIR injury applications [110]. In vitro studies revealed a tri-phase pattern with an initial burst release of ~30% within the first 24 h, followed by a slow, sustained release of up to ~44%. A pre-treatment model was used to determine the effectiveness of the Cur-PEG-PLGA NPs, where rats were fed the NC. Following the induction of CIR, histopathological slices stained using H&E showed that NC protected neurons significantly against injury, whereas free curcumin was less protective, as visualized from the appearance of the neuronal cells. Additionally, pre-treatment with NC increased mitochondrial membrane microviscosity (visualized using fluorescence depolarization), minimized ROS production (observed via 2′,7′-dichlorofluorescein diacetate (DCFDA), and significantly protected neurons from CIR-stimulated apoptosis, as Western blotting revealed decreased Bcl-2 levels. This study effectively demonstrates that the oral drug delivery of NCs has the potential to be therapeutic against CIR following a stroke, and may also be beneficial in other age-related neurological disorders.

#### 4.3.4. Subarachnoid Hemorrhage Model

Zhang et al. observed that poly(lactide-co-glycolide) (PLGA)-encapsulated curcumin nanoparticles (Cur-NPs) exerted neuroprotective effects following early brain injury in a rat subarachnoid hemorrhage (SAH) model [70]. In this study, the emulsification/solvent diffusion nanoparticle synthesis method was utilized. Drug release studies utilizing dialysis demonstrated a dual-release profile, where 71.7 ± 4.1% of curcumin was freed in the first 12 h, followed by a sustained delivery of up to 85.1 ± 3.5% 36 h later. Induction of SAH in Sprague-Dawley rats occurred using intracranial endovascular perforation, where a suture was rapidly inserted and removed from an artery to allow reperfusion. Twenty-four hours after SAH, results demonstrated that both curcumin and Cur-NPs exhibited neuroprotective effects, decreased brain-water content, and diminished blood–brain barrier permeability by reducing VEGF and MMP9 expression, thus increasing the expression of claudin-5, occludin, and ZO-1. Additionally, using DCDHF and LDH assays, Cur-NPs were found to reduce oxidative stress and reduce apoptosis in the SAH-induced rats. The results of this study reveal that both free curcumin and Cur-NPs were effective in providing neuroprotection following an induced subarachnoid hemorrhage. However, the results demonstrate that curcumin-containing nanoparticles improved curcumin’s bioavailability by at least 10-fold. Additionally, the Cur-NPs reduced the COX-2 and iNOS inflammatory markers and IL-1β, IL-6, and TNF-α proinflammatory cytokines, as examined with an ELISA assay.

Similarly, Chang et al. investigated the efficacy of nanocurcumin on a SAH-induced early brain injury model in Sprague-Dawley rats [111]. The nanocurcumin used was created using PLGA, which was then emulsified with PVA, to improve bioavailability. Following SAH induction, it was observed that nanocurcumin improved motor functions in a dose-dependent manner, calculated by the motor deficiency index, and substantially decreased the paraplegia rate. Analysis of the cerebrospinal fluid (CSF) showed that nanocurcumin exhibited anti-inflammatory effects, as observed by the reduction in NF-ĸB expression by Western blot, and decreased mRNA expression levels of IL-1β, IL-6, and TNF-α when compared to the SAH groups. Finally, mitochondrial caspase-3 and -9a, determined by rt-PCR, were significantly reduced, preventing the production of reactive oxygen species. These findings elaborate on and confirm the findings from the study presented by Zhang and colleagues.

#### 4.3.5. Traumatic Brain Injury Model

Lastly, Narouiepour et al. investigated the effects of a combination of neural stem cells (hNS/PCs) and curcumin-loaded noisome nanoparticles in a Wistar rat model of traumatic brain injury [112]. The curcumin-loaded noisome nanoparticles (CM-NPs) in this study were prepared using a thin-film hydration method to encapsulate curcumin into the shell of the nanoparticles. Following traumatic brain injury (TBI) and a 10-day oral administration of CM-NPs, both the CM-NPs and human neural stem/progenitor cells (hNS/PCs) + CM-NPs treatment groups demonstrated significantly reduced brain water content in the cerebrum compared to the no treatment control group. Additionally, immunohistochemistry revealed there was a significant reduction in inflammation, as demonstrated by a reduction in GFAP-positive cells in the hNS/PCs + CM-NPs group and a decrease in the mean number of Iba-1+ cells in the CM-NPs group. Furthermore, TNF-α+ cells were significantly decreased in the curcumin groups. From the results, it was observed that the combination of neural stem cell therapy with curcumin nanoparticles showcases neuroprotective and anti-inflammatory properties that may be potential theraperutics following TBI. Curcumin delivery from nanoparticle biomaterials is summarized in Table 3.

## 5. Curcumin Delivery from Hydrogels

Hydrogels are three-dimensional (3D) networks of hydrophilic polymers that maintain their structure via chemical and/or physical cross-linking. Hydrogels are ideal candidates for drug-delivery applications due to their highly tunable properties, ability to encapsulate and protect drugs, and controlled degradation [113]. Specifically, controlled drug release is determined by the amount of physical or chemical cross-linking in the hydrogel, as these parameters determine porosity and the likelihood of the hydrogel swelling in the aqueous environment, where increased swelling promotes increased drug release [114]. Finally, hydrogels are one of the most common scaffolds used in tissue engineering due to their ability to mimic the porous ECM found in many tissues, as well as provide cellular via attachment ligands [115].

### 5.1. Skin Tissue Engineering

#### 5.1.1. Full-Thickness Wound Models

Wathoni et al. created a curcumin-containing 2-hydroxypropyl-γ-cyclodextrin (Cur/HP-γ-CyD) complexed in a sacran (derived from Aphanothece Sacrum cyanobacteria)-based hydrogel film (Sac-HGF) [71]. Curcumin release data from the Cur/HP-γ-CyD complex indicated a biphasic curve, with 49.69 ± 3.74% initial release occurring within the first 24 h, followed by a gradual release up to 69.49 ± 5.16% until 120 h. Using a gravimetric technique with these hydrogel films, Wathoni and colleagues observed lowered re-swelling ability in the curcumin-hydrogel films compared to Sac-HGF alone but noted that there was still a high re-swelling ability. Antioxidant activity was observed in NIH3T3 fibroblast cells, where application of the Cur/HP-γ-CyD complex in Sac-HGF significantly improved cell viability when treated with H2O2. Wound healing properties were analyzed in a full-thickness excisional wound model in hairless mice. Upon application, it was noted that treatment with Cur/HP-γ-CyD significantly improved wound repair ability by increasing wound contraction at 3, 7, and 14 days compared to the no-treatment control group.

Another study, published by Zhou et al., loaded curcumin into 2-(methacryloyloxy) ethyl 2-(trimethylammonio) ethyl phosphate copolymers (P(MPC-co-GMA)), which were then synthesized into a hydrogel wound dressing using ultrasonication and tube inversion methods (Cur-gel-G10M20) [116]. Similar to previous studies, the hydrogel used in this paper exhibited antibacterial effects against *S. aureus* and *E. coli*. Additionally, utilizing fluorescence spectrometry procedures, a sustained release was observed with Cur-gel-G10M20, where the cumulative release was 15% at 24 h and 48.5% at 192 h. When applied to a full-thickness injury model in Sprague-Dawley rats, the curcumin-loaded hydrogel exhibited wound closure after 14 days, with a faster rate being potentially due to curcumin’s anti-inflammatory and antioxidant properties compared to the 92.4% wound closure observed in the saline-only control group. Lastly, as observed in H&E images, the curcumin-loaded and control hydrogel groups promoted the reconstruction of hair follicles due to an increase of CD31 expression in the treatment groups, while the controls did not promote epidermal appendage reconstruction.

Gong et al. utilized a full-thickness wound model to examine the effects of a hydrogel construct containing polymeric micelle-encapsulated curcumin (Cur-M-H) [117]. With a drug loading capacity and encapsulation efficiency of the curcumin-loaded polymer at 14.76 ± 0.12% and 98.40 ± 0.81%, respectively, high-performance liquid chromatography (HPLC) demonstrated a cumulative curcumin release of 40.1 ± 2.5% on day 14. Although the Cur-M-H composite had a lower curcumin delivery than the Cur-M micelles alone, the researchers indicated that this delayed release could lead to more sustained drug delivery in future applications. With that, Cur-M-H exhibited increased wound healing in vivo, demonstrated by a thicker epidermis with MT staining, as well as increased tensile strength in a linear incision model when compared to the Cur-M control on day 14. Additionally, the hydrogel composite promoted greater wound closure in an in vivo full-thickness excision wound model than the Cur-M group. Furthermore, in both wound models, MT staining indicated Cur-M-H groups had better granulation and higher collagen content and showed great promise for wound healing applications.

#### 5.1.2. General Wound Healing Models

Albarqi et al. developed curcumin-loaded chitosan and sodium alginate membranes using microwave-based physical cross-linking to promote wound healing following skin injuries [64]. With microwave treatment, when compared to the untreated controls, the membrane’s swelling ability was significantly increased, while the drug release that was quantified using HPLC was extended to 41 ± 4.2% within one day, with an initial loading rate of 87.6 ± 5.2%. Additionally, in a full-thickness open incision wound healing model on male Sprague-Dawley rats, there was significantly faster wound closure and a higher percentage of re-epithelialization in groups that received the microwave-treated curcumin-loaded membranes after 14 days compared to the controls where curcumin was administered alone or animals that did not receive any treatment. These results suggest that the microwave-treated hydrogel affected skin re-epithelialization more than the controls due to the induction of polymer-drug interactions.

Additionally, Niranjan et al. observed that TiO2-Curcumin nanocomposites integrated into polyvinyl alcohol (PVA)/chitosan hydrogel patches increased the rate of wound healing in Wistar rats [73]. Using UV-vis, the constructs demonstrated a dual-release profile, with an initial burst of curcumin delivery (~80% after ~2.5 days) followed by sustained release over 25 days (~100%). The patches also possessed antibacterial properties. When tested against both gram-positive and gram-negative bacteria commonly found at wound sites, it was observed that the patches created inhibition zones. For Bacillus subtilis, Staphylococcus aureus, Escherichia coli, and Pseudomonas aeruginosa, the inhibition zones measured 14 mm, 15 mm, 18 mm, and 20 mm, respectively. When measuring the amount of hydroxyproline utilizing a hydroxyproline assay kit, where higher values indicate faster tissue regeneration, the PVA/Chi/Cur patch group produced more hydroxyproline than the commercial ointment and untreated groups. Furthermore, when analyzed via histological imaging following application to a Wistar rat full-thickness wound model, increased hair growth and restoration of the dermal region were observed 16 days after wound creation. Additionally, no inflammation or necrosis was observed in animals treated with the PVA/Chi/Cur patch.

Finally, Singh et al. used a curcumin-embedded decellularized goat small intestine submucosa (DG-SIS) hydrogel to mitigate the oxidative stress that occurs at wound sites [118]. While evaluating the scaffolds’ hydrophilicity, it was observed that there was an inverse relationship between curcumin amount and water retention, where the lowest water retention was observed in the group with the highest curcumin concentration (DG-SIS/C3). Contrastingly, the scaffolds with the highest amount of curcumin incorporation had increased degradation rates, where DG-SIS/C3 degraded within 96 h, while the DG-SIS/C2 and C1 scaffolds degraded within 105 h and 120 h, respectively. Finally, the DG-SIS/C3 scaffold exhibited the highest biocompatibility, free-radical scavenging abilities, and cellular proliferation.

#### 5.1.3. Diabetic Wounds

Curcumin-loaded hydrogels have also been used to promote wound healing in a streptozotocin (STZ)-induced diabetic rat model. Shah et al. created a curcumin-loaded hyaluronic-acid-Pullulan-g-F127 injectable hydrogel (CUR-HA-Pu-g-F127) [74]. Following loading of the hydrogel with 5 mg/mL curcumin and release analysis with ultraviolet-visible spectrophotometry (UV-vis), it was determined there was little initial burst release, with 50% curcumin being freed within eight hours. Additionally, a sustained delivery of up to ~84% was observed over 24 h. Following in vivo application to a biopsy punch wound in adult male Wistar rats, the hydrogel improved diabetic wound healing by reducing the inflammatory response, as seen by H&E and MT staining. Furthermore, dermis formation was much more organized and presented in a well-layered pattern, with greater collagen regeneration when compared to the unorganized dermis of the saline-only controls.

Similarly, using a hydrogel with curcumin conjugated with hyaluronic acid (HA-Cur), Sharma et al. observed that hydrogel application to STZ-induced diabetic Swiss Albino wounds led to increased wound healing in vivo [119]. Interestingly, when applied to in vitro human keratinocyte (HaCaT) cells, Sharma and colleagues observed a decrease in wound healing in the curcumin-treated groups, which they attributed to an inhibition of cellular migration from curcumin. However, opposite effects were observed in vivo when applied to STZ-induced diabetic mice. Following topical application of their hydrogel, the HA-Cur group, containing 25 µM curcumin, outperformed the untreated, curcumin-treated, and HA-treated control groups with a total wound closure of ~96% after 14 days. Furthermore, H&E staining showed a well-formed dermal layer with complete re-epithelialization. Additionally, the HA-Cur construct exhibited anti-bacterial efficacy, which is attributed to curcumin’s inherent antibacterial properties.

#### 5.1.4. Burn Wounds

Following severe burn injuries, edema, inflammation, and scarring frequently remain at the injury site. To combat these effects, El-Rafaie et al. created curcumin-loaded gel-core hyalurosomes (Curc-GC-HS) [75]. Utilizing dialysis and UV-Vis methods, at the 2- and 6-h time points, the cumulative release was noted to be ~50% and ~81%, respectively. For this study, a burn wound was created on the backs of female Sprague-Dawley rats, which were chosen due to their similar epidermal structure to humans. By day ten, Curc-GC-HS exhibited improved wound healing abilities (99.7% ± 0.34) with complete healing and minor-to-no scarring compared to saline controls. Furthermore, a normal epithelial structure with woven collagen fibers was visualized with H&E staining.

Similarly, Dang et al. observed greater epidermal regeneration and increased collagen density following the application of a curcumin-loaded chitosan-g-pluronic copolymer nanocomposite hydrogel (nCur-CP) to a burn wound model in male Albino mice [120]. When investigated for its antibiotic abilities against commercially available antibiotics, the nCur-CP hydrogel was more effective against gram-positive bacteria, such as *P. aeruginosa*, *S. aureus*, and *E. coli*, which could be due to chitosan’s ability to disrupt the membranes’ surface charges. Additionally, when visualized in a second-degree burn model in vivo with histological staining on day 14, the nCur-CP group demonstrated increased collagen formation and a thicker epidermis than the no-treatment and standard-treatment (burn cream) control groups. Furthermore, the curcumin group also exhibited greater wound closure in a third-degree burn model after 22 days. Finally, due to curcumin’s ability to promote angiogenesis, the mice who received nCur-CP hydrogel treatment showed a significant increase in blood vessel growth rate and density.

### 5.2. Bone Tissue Engineering

Current osteosarcoma treatments encompass a combination of both surgical resection and radiotherapy or chemotherapy. Although these methods have improved outcomes in patients with osteosarcoma, large bone defects that result from cancer may remain following chemotherapy and radiotherapy treatment, in addition to the systemic damages caused by the intense treatment. To improve regeneration outcomes, Tan et al. designed a minimally invasive hydrogel to target osteosarcoma therapy and bone regeneration [76]. UV-Vis Spectroscopy revealed differing curcumin release rates between the constructs: the Cur-MPs gel demonstrated ~20% release after 70 h, while the Cur-MPs/IR820 Gel + Laser construct demonstrated ~32% release after ~200 h. Utilizing an injectable curcumin-microsphere/IR820 co-loaded hybrid methylcellulose hydrogel (Cur-MP/IR820 gel) platform and localized hyperthermia for a tumor model in female Balb/c mice, an inbred Albino strain mainly utilized in immunology and cancer research, investigators were able to increase curcumin delivery and cell membrane permeability upon irradiation with a laser, leading to increased tumor cell death compared to the no-treatment, MC-gel, Cur-MP solution, and Cur-MP/IR820 gel groups. Additionally, using near-infrared imaging and body weight calculations in an in-situ bone tumor model, researchers observed that the Cur-MP/IR820 hydrogel had delayed tumor growth compared with the Cur-MP group. Therefore, the combination of the curcumin-loaded hydrogel and laser treatment demonstrated the best therapeutic effect, with reduced invasiveness of cancer into surrounding bone tissue.

### 5.3. Central Nervous System Engineering

#### 5.3.1. Spinal Cord Injury

Following spinal cord injury, acute inflammation leads to neuronal apoptosis, the development of glial scarring, and the production of reactive oxygen species, leading to difficulties in neuronal regeneration and repair. Current treatments for spinal cord injuries focus on mitigating this unfavorable microenvironment that develops upon damage. Luo et al. created a hybrid hydrogel containing Fluorenylmethyloxycarbonyl protecting group (Fmoc)-grafted chitosan (FC) and Fmoc peptide (FI), which, when combined, have injectable and self-healing properties [77]. Following the combination with curcumin to form an FC/FI-Cur hydrogel and visualization with UV-Vis, the hydrogel exhibited a curcumin burst-release within 48 h (~68%), with a more sustained release of ~82% over 168 h. Luo et al. utilized NF200+ and S100+ (neural and Schwann cell markers) staining and performed statistical analysis on neurite length, the number of Schwann cells migrating as a function of distance, as well as the percentage of axon-attached Schwann cells, and compared the experimental group to FC-only and FC/FI controls. Immunostaining and confocal microscopy revealed FC/FI-Cur accelerated neurite outgrowth from dorsal root ganglion (DRG) neurons in vitro while enhancing Schwann cell (SC) migration away from the DRG body, leading to enhanced remyelination of regenerated nerves. Furthermore, when applied to an in vivo Sprague-Dawley rat transection model of spinal cord injuries, immunostaining revealed modulation of the ARG1+/CD68+ inflammatory responses as well as increased SC migration and remyelination after 14 days.

In an effort to promote functional recovery following chronic SCI, Elkhenany et al. utilized a combinatorial approach comprising a hyaluronic acid containing polypyrrole-coated fibers peptide hydrogel (PM), human-induced neural progenitor cells (iNPCs), and nanocurcumin, abbreviated as PM-embedded iNPCs with CURC [121]. Following in vitro application to organotypic spinal cord slices, the curcumin construct promoted the formation of neuron-like processes from iNPCs when compared to the PM-iNPC control, demonstrated by an increase in GFP staining. When applied to a contusive in vivo SCI model in Sprague-Dawley rats, neuropreservation was analyzed by examining the expression of β-III-tubulin-positive neuronal fibers, where implantation of the fiber-containing (PPY)-PM-iNPC-CURC scaffold maintained expression, while the PM-only, PPY-PM-CURC, PM-iNPCs, PM-CURC-iNPCs, and PPY-PM-iNPCs controls decreased expression. Furthermore, PPY-PM-iNPC-CURC decreased scar tissue formation, as seen by the lack of GFAP staining compared to the control groups.

Similarly, Requejo-Aguilar et al. examined a construct involving ependymal stem/progenitor cells of the spinal cord (epSPCs) and a polyacetal-curcumin conjugate (PA-curcumin) [63]. HPLC quantification of samples after ~180 h demonstrated a ~100% release from the construct at a pH of 5.5 and ~50% release at a pH of 6.5. Following in vitro work on epSPCi co-cultures with DRG, their construct not only induced neuron survival but also promoted increases in axonal length and greater axonal sprouting, as seen by increases in Tau and/or MAP2 immunostaining, which they attribute to Rho/Rock kinase inhibition. These effects also persisted following visualization in a severe contusive Sprague-Dawley rat spinal cord injury model. A decrease in GFAP expression indicated a reduction in astrogliosis, compared to the PA-only control, as well as a decrease in the inflammatory response. Functional recovery with the 21-point BBB locomotion scale was examined in an in vivo chronic state, which was enhanced only when combined with epSPCs, indicating that a combinatorial approach may be more promising than a biomaterial approach alone.

#### 5.3.2. Traumatic Brain Injury

One component that contributes to the harsh environment in central nervous system injuries is the development of reactive oxygen species from blood–brain barrier disruption, which leads to increased neuronal death [78]. To mitigate these effects, Qian et al. embedded curcumin into a matrix-metalloproteinase (MMP)-responsive triglycerol monostearate (TM) hydrogel (TM/P) (TM/PC). Characterization of the hydrogel utilizing UV-vis revealed a curcumin release rate of more than 80% in patient-derived cerebrospinal fluid (CSF) by day 14. Further characterization demonstrated ROS and/or MMP activity influence on the curcumin release rate from the construct; curcumin delivery was contingent on the absence or presence of different MMPs and reactive oxygen species. An in vivo TBI model was utilized with 5-week-old Albino ICR mice, which are an outbred strain frequently used in toxicology and pharmacology studies. Twenty-one days post-TBI induction, the construct successfully mitigated ROS (measured by DCFH-DA assay), maintained BBB integrity (tested with EB extravasation), and lessened brain edema (detected by T2 MRI), indicating that it decreased secondary damage caused by ROS compared to TM/C and TM/P controls. Additionally, neuroinflammation was reduced, demonstrated by a decrease in GFAP and Iba-1 expression, as well as M1 macrophage polarization. Finally, the detection of the DX and GAP43 proteins indicated that the TM/PC hydrogel facilitated neuroregeneration and recovery at the TBI site. Curcumin delivery from hydrogel biomaterials is summarized in Table 4.

## 6. Discussion

With its innate antioxidant, anti-inflammatory, and anti-microbial properties, curcumin currently demonstrates great promise for use in biomedical engineering applications. Despite its many advantages for tissue engineering, its highly hydrophobic nature hinders its bioavailability, making it difficult to use in clinical applications. Curcumin delivery from biomaterials is one promising solution to this limitation and allows for local drug delivery to injured or diseased tissues. The studies presented in this review have all demonstrated beneficial outcomes from employing biomaterial carriers, with many utilizing in vivo models. However, one limitation was the use of different mouse models that are predisposed to the development of immunogenic responses or harmful phenotypes. Thus, although all models presented displayed similar results, a generalized mouse model should be used to prevent possible skewing of the results. One other drawback to the use of curcumin biomaterials, specifically nanoparticles, is that they are not diseased-cell- or tissue-specific; therefore, investigators should explore methods to add mechanisms to target specific tissues.

An exciting avenue for future research is the use of tissue-specific stem cells in combination with curcumin—in aggregate, the studies reviewed herein have shown great potential for this approach. Finally, it is imperative to determine the safety of higher curcumin doses with biomaterial delivery, as DNA damage is known to occur with high doses of curcumin. Future studies should determine the dosage limit, especially from biomaterial delivery, as they are more potent due to higher bioavailability.

Several studies have examined the use of electrospun fiber-encapsulated hydrogels with curcumin for central nervous system injuries, and future work can examine the use of electrospun fibers alone for CNS applications. Curcumin has demonstrated an innate ability to mitigate acute injury symptoms, specifically ROS depletion and anti-inflammatory responses, but could be researched in dual-drug delivery systems, where an additional drug acts in combination with curcumin to promote axon regeneration and functional recovery. One example of a proposed construct is core–shell electrospun fibers, which would allow for dual drug delivery while providing the necessary scaffolding for neural tissue regeneration. Additionally, if released over a long duration, curcumin could be utilized to combat CNS acute and secondary injury effects. Pro-drugs are chemical derivatives of a bioactive molecule that are modified to improve a pharmacological property, which are then metabolized into the active drug. Polymerized pro-drugs (poly(pro-drugs)) have become of recent interest, as pro-drugs can be incorporated into a polymer backbone to allow for controlled and tunable delivery of the species. Chen et al. have demonstrated the ability to create tunable poly(pro-curcumin) compounds, each containing different amounts of curcumin, with a PEG backbone to increase hydrophilicity [122]. One specific formulation, P50, exhibits a dual-release profile, which has the potential to mitigate ROS over long durations, increasing neuronal survival upon injury. Future work can investigate the translation and incorporation of poly(pro-compounds) into scaffolds such as electrospun fibers or hydrogels.

Overall, researchers should supply the exact concentration of curcumin being released from their constructs. Many studies provide information such as drug loading efficiencies, encapsulation efficiencies, and release rates; however, knowing the concentration needed to achieve their therapeutic result is imperative for clinical translation. Furthermore, the researchers should divulge if the release rate was determined by controlled release from the biomaterial. If not, and the constructs did not undergo a wash step before quantification to remove curcumin floating on the surface, then the loading efficiency and release rate reported may not be accurate.

Finally, as future work utilizing curcumin continues, it is imperative that lead-free samples are used. Forsyth et al. collected samples of turmeric from nine major districts in Bangladesh and found evidence that lead chromate pigments were present in samples from seven districts [123]. Although turmeric is naturally yellow, consumers prefer a strong yellow pigment; thus, distributors adulterate the color by adding lead chromate. Increased lead exposure results in a myriad of health issues; therefore, turmeric should undergo lead testing to ensure that it, and the curcumin that is derived from it, do not bring harm to the public.

## 7. Conclusions

This review first discusses the reactivity of curcumin, its beneficial role in medicine throughout history, and the limitations it faces in current applications. It then examines various biomaterial and composite methods to effectively deliver curcumin to an injury or disease site, from electrospun fibers to hydrogel scaffolds, while analyzing a multitude of tissue engineering applications. Throughout the previously mentioned studies, it has been noted that curcumin biomaterials enhance wound healing and angiogenesis in skin wound models, promote bone reformation following bone defects, and exhibit neuroprotective and neuroregenerative effects in the nervous system. Although these effects could likely be accomplished with curcumin in its standard form, high doses would be necessary. Curcumin delivery through biomaterial systems provides targeted delivery and much greater bioavailability with lower doses, allowing researchers to fully utilize its beneficial properties.

## Figures and Tables

**Table 1:** Advantages and disadvantages of analytical quantification methods for curcumin.

Method	Lowest Detection Limit [59]	Advantages	Limitations	Release Mediums	References
High-Performance Liquid Chromatography (HPLC)	15 ng/mL	- Detect individual curcuminoids - High accuracy and reliability	- High flow rate - Longer run time - High limit of detection	PBS	[60,61,62,63]
Ultra-High-Performance Liquid Chromatography (UHPLC)	0.3 ng/mL	- Increase in resolution per time - Cost-effective - Rapid analysis	- Higher maintenance cost	- Acetonitrile/acetic acid (80/20)	[64]
Ultraviolet-visible Spectrophotometry (UV-Vis)	39 ng/mL	- Rapid analysis - High sensitivity	- Low precision - Individual curcuminoid composition not given	PBS; Water/Ethanol; Saline/Ethyl Alcohol	[65,66,67,68,69,70,71,72,73,74,75,76,77,78]

**Table 2:** Curcumin delivery from electrospun fiber biomaterials.

Ref.	Biomaterial Type(s)	Curcumin Incorporation Method	Electrospinning Parameters	Curcumin Release Kinetics	Model(s)	Significant Finding(s)
[60]	Poly(ε-caprolactone)-poly(ethylene glycol)-poly(ε-caprolactone) (PCEC) Fibers	Blending	- Flow Rate: 6 mL/h - Voltage: 18 kV - Distance (Nozzle to Collector): 12 cm	*After 10 days:* - 5%-Cur/PCEC: 52.7% - 10%-Cur/PCEC: 66.4% - 15%-Cur/PCEC: 78.5% - 20%-Cur/PCEC: 87.2%	- Adult Female Wistar Rats - Full-Thickness Dermal Defect Model	PCEC/Curcumin fibrous mats significantly increased wound closure compared to the untreated control
[65,87]	Poly(ε-caprolactone)/gum tragacanth (PCL/GT/Cur) Fibers	Blending	- Flow Rate: 1 mL/h - Voltage: 15 kV - Distance (Nozzle to Collector): 15 cm	*After 20 days,* - PCL/GT/3%-Cur: 65% Release	- Male Diabetic Sprague-Dawley Rats - Wound Healing Model	PCL/GT/Cur increased wound closure with well-formed granulation tissue
[88]	Poly(ε-caprolactone) (PCL) Fibers	Blending	- Flow Rate: 2 mL/h - Voltage: 25 kV - Distance (Nozzle to Collector): 10 cm	*After 3 days,* - 3%-Cur/PCL: ~20 µg - 17%-Cur/PCL: ~35 µg	- Male Diabetic C57/B6 Mice - Wound Healing Model	17%Cur-PCL fiber application accelerated wound closure and decreased inflammation compared to PCL nanofiber control
[91] *	Asymmetric Membrane: Collagen nanofiber and PLGA-aspirin nanoparticles (PACNFs); Curcumin and collagen nanofibers (CCNFs)	Blending	- Flow Rate: 1.5 mL/h - Voltage: 20 kV - Distance (Nozzle to Collector): 10 cm	-	- Adult Mongrel Dog - Alveolar Bone Defect Model	Overall composite material promoted new bone and soft tissue formation compared to commercial control

* Denotes a composite scaffold, where two or more biomaterial platforms are combined.

**Table 3:** Curcumin delivery from nanoparticle biomaterials.

Ref.	Biomaterial Type(s)	Preparation Method	Drug Loading %	Encapsulation Efficiency %	Delivery Method	Curcumin Release Kinetics	Model(s)	Significant Finding(s)
[61]	Curcumin-loaded PLGA (PLGA-CC)	O/W Emulsion-Solvent Evaporation Technique	-	89.2 ± 2.5%	Intradermal	*After 8 days,* 75.7 ± 3.4%	- Female RjHan:NMRI Mice - Full-Thickness Excisional Wound Model	PLGA-CC promoted greater re-epithelialization and two-fold higher wound healing compared to control
[66] *	Electrospun PCL and Gelatin scaffold containing curcumin-loaded chitosan nanoparticles (PCL/Gela/NCs/Cur)	Solvent Evaporation	4.2 ± 0.2%	93 ± 5%	Topical	*After 4 days*, 100% (NCs/Cur) *After 10 days,* ~83% (PCL/Gela/NCs/Cur)	- Male Wistar Rats - Full-Thickness Excisional Wound Model	PCL/Gela/NCs/Cur demonstrated higher re-epithelialization, collagen synthesis, and wound healing
[99]	Curcumin-loaded hyalurosomes; curcumin-loaded liposomes	-	-	-*Hyalurosomes:* ~79% -*Liposomes*: ~66% (~54% after leakage)	Topical	-	- Female CD-1 Mice - Wound Healing Model	Curcumin-loaded hyalurosomes reduced inflammation, edema, MPO activity, and promoted re-epithelialization
[102] *	Cellulose nanocrystals loaded chitosan films with curcumin/silver nanoparticles	-	-	-	Topical	-	- Albino Wistar Rats - Full-Thickness Excisional Wound Model	Combination of curcumin with Ag nanoparticles greatly improved wound healing compared to curcumin alone
[62] *	Curcumin-loaded polyvinyl alcohol/collagen composite films (CPCF)	Solvent Evaporation	9.61 ± 0.12%	96.09 ± 1.21%	Topical	*After 5 days*, 90% (Cur NPs) 76% (CPCF)	- Male Sprague-Dawley Rats - -Full-Thickness Wound Model	CPCF treatment increased wound healing and epithelialization, as well as promotion of hair follicles
[107]	Curcumin-loaded nanomicelles (gold nanorods [GNRs]-1/curcumin in polymeric nanomicelles) (GNRs-1/curc@PMs)	Double Re-emulsification	-	41%	Injection at tendon	-	- Sprague-Dawley Rats - Tendon Rupture and Repair	GNRs-1/curc@PMs reduced peritendinous adhesions and demonstrated greater tendon strength with laser exposure
[108]	Curcumin-loaded nanomicelles (commercially available: SinaCurcumin)	-	-	-	Oral Gavage (100 mg/kg/day)	-	- Male Sprague-Dawley Rats - Partial Transection of Gastrocnemius Muscle	Curcumin-loaded nanomicelles had increased angiogenesis and muscle fiber regeneration following laceration injury
[109]	Curcumin-poly(ε-caprolactone) nanoparticles (Cn-NPs)	Single Emulsion-Solvent Evaporation	-	-	Intraperitoneal Injection	-	- Male Wistar Rats - Glycerol Induced Muscle Injury	Cn-NPs reduced inflammation, decreased muscle fibrosis, and enhanced muscle regeneration following muscle injury
[67]	Curcumin-encapsulated hyaluronic acid-palmitate nanoparticles (Cur-HA-palmitate NPs)	Emulsification/Solvent Diffusion	-	-		*After 72 h,* ~70%	- Mouse Striatal knock-in cells expressing mutant huntingtin	Cur-HA-palmitate NPs had greater cell penetration and reduced susceptibility to apoptosis
[68]	Curcumin-encapsulated solid lipid nanoparticles (C-SLNs)	Solvent Evaporation	93.25 ± 1.85%	-	Oral Gavage (40 mg/kg/day)	*After 6 h,* 53.77 ± 2.45%	- Female Wistar Rats - 3-NP-Induced HD	C-SLNs increased mitochondrial activity, increased locomotor activity and reduced gait abnormalities
[69]	Curcumin-encapsulated PLGA nanoparticles (Cur-PLGA-NPs)	Emulsion-Solvent Evaporation	-	~77 ± 5%	Intraperitoneal Injection	*After ~36 h,* ~60% *After 7 days,* ~74%	- Wistar Rats - Alzheimer’s Disease Model	Cur-PLGA-NPs show greater reversal AD dysfunction via activation of Wnt/β-catenin pathway
[110]	Curcumin-incorporated PEGylated PLGA nanoparticles (NC)	Modified Emulsion-Diffusion-Evaporation	-	58.9 ± 8.67%	Oral Gavage	*After 24 h*, ~30% *After 48 h,* ~44%	- Female Sprague-Dawley Rats - Cerebral Ischemia-Reperfusion	NC pre-treatment to CIR model had neuroprotective effects by reducing ROS-mediated damage and apoptosis
[70]	Poly(lactide-co-glycolide) (PLGA)-encapsulated curcumin nanoparticles (Cur-NPs)	Emulsification-Solvent-Diffusion	-	81.7 ± 4.6%	Intraperitoneal Injection	*After 12 h*, 71.7 ± 4.1% *After 36 h,* 85.1 ± 3.5%	- Male Sprague-Dawley Rats - Subarachnoid Hemorrhage Model	Cur-NPs attenuated blood-brain barrier dysfunction and glutamate concentrations, and reversed SAH-induced apoptosis
[111]	Curcumin-encapsulated PLGA nanoparticles	Two-step nanoprecipitation	-	-	Injection	-	- Male Sprague-Dawley Rats - Double Subarachnoid Hemorrhage Model	Nanocurcumin decreased inflammation and reduced caspase-9 expression
[112]	Curcumin-loaded noisome nanoparticles	Thin-film hydration	-	-	Oral Gavage	-	- Male Wistar Rats - Traumatic Brain Injury Model	CM-NPs combined with human neural stem/progenitor cells reduce brain edema and reduce inflammation

* Denotes a composite scaffold, where two or more biomaterial platforms are combined.

**Table 4:** Curcumin delivery from hydrogel biomaterials.

Ref.	Biomaterial Type(s)	Curcumin Incorporation Method	Delivery Method	Curcumin Release Kinetics	Model(s)	Significant Finding(s)
[71] *	Curcumin/2-hydroxypropyl-γ-cyclodextrin (HP-γ-CyD) complex in sacran-based hydrogel	Water Casting	Topical	*After 24 h,* 49.69 ± 3.74% *After 120 h,* 69.40 ± 5.16%	Hairless Mice - Two Full-Thickness Excisional Wounds	High elastic modulus, Cur/HP-γ-CyD complex in Sac-HGF increased wound healing ability
[116]	Curcumin-loaded 2-(methacryloyloxy) ethyl 2-(trimethylammonio) ethyl phosphate copolymer (P(PC-*co*-GMA)) hydrogel (Cur-gel-G10M20)	Used as buffer solution in hydrogel preparation	Topical	*After 24 h*, 15% *After 192 h*, *48.5%*	Sprague-Dawley Rats - Full-Thickness Skin Defect Model	Cur-P(MPC-*co*-GMA) hydrogel increased wound healing rate and promoted reconstruction of hair follicles
[117] *	Curcumin-loaded micelles in a thermosensitive PEG-PCL-PEG hydrogel composite (Cur-M-H)	One-step solid dispersion into PEG-PCL copolymer	Topical	*After 14 days,* 40.1 ± 2.5%	Male Sprague-Dawley Rats - Linear Incision Wound Model - Excision Wound Model	In both wound models, Cur-M-H increased collagen formation, better granulation, and greater wound repair
[64]	Curcumin-loaded chitosan-sodium alginate hydrogel membrane	Dissolved into hydrogel solution	Topical	*After 24 h,* 41 ± 4.2% (Microwave-crosslinked)	Male Sprague-Dawley Rats - Full-Thickness Open Incision Wound	Microwave-treated membrane promoted greater re-epithelialization with increased collagen deposition and greater epidermal definition
[73] *	Polyvinyl alcohol/sodium alginate/titanium dioxide-curcumin patch (PVA/SA/TiO_2_-Cur)	Synthesized to TiO_2_ to form a nanocomposite	Topical	*After ~2.5 days,* ~80% *After 25 days,* ~100%	Wistar Albino Rats - Full-Thickness Wound Model	PVA/SA/TiO_2_-Cur patch application increased wound healing and exhibited anti-bacterial properties against gram-positive and -negative bacteria
[118]	Curcumin-embedded decellularized goat small intestine submucosa (DG-SIS) hydrogel	Dissolved into scaffolds	-	DG-SIS/C3 *After 5 h*, 24% *After 96 h,* 73%	-	DG-SIS/C3 exhibited the greatest antibacterial properties, increased radical scavenging, and good biocompatibility
[74]	Curcumin-loaded hyaluronic-acid-Pullulan-g-F127 hydrogel (CUR-HA-Pu-g-F127)	Mixed into hyaluronic acid	Subcutaneous Injection	*After 8 h*, 50% *After 24 h,* ~84%	- Male Diabetic Wistar Rats Biopsy Punch Wound	CUR-HA-Pu-g-F127 increased rate of wound healing and closure
[119]	Curcumin-conjugated hyaluronic acid hydrogel (HA-Cur)	Mixed into hyaluronic acid	Topical	-	- Male Swiss Albino Mice Full-Thickness Wound Model	HA-Cur revealed antibacterial properties, decreased oxidative damage, and increased wound healing
[75]	Curcumin-loaded gel-core hyalurosomes (Cur-GC-HS)	Gelled into Pluronic F-127	Topical	*After 2 h,* ~50% *After 6 h,* ~81%	Female Sprague-Dawley Rats - Burn Wound Model	Cur-GC-HS increased wound healing with no scar formation, as well as higher skin deposition
[120]	Curcumin-loaded chitosan-g-pluronic copolymer nanocomposite hydrogel (nCur-CP)	Mixed into CP	Injection	-	Male Albino Mice - Second and Third Degree Burn Wound Model	nCur-CP enhanced wound closure, increased collagen density, thicker epidermis formation, and better granulation
[76] *	Curcumin-microsphere/IR820 coloaded hybrid methylcellulose hydrogel (Cur-MP/IR820)	Encapsulated into PLGA microspheres	Injection	- Cur-MPs Gel *After 70 h*, ~20% - Cur-MPs/IR820 Gel + Laser *After ~200 h*, ~32%	- Female Balb/c Mice Tumor Plague Embedment Approach Model	Cur-MP/IR820 exhibited thermal-accelerated curcumin release and increased tumor cell apoptosis, osteogenic properties increasing bone reconstruction
[77]	Curcumin-loaded Fluorenylmethyloxycarbonyl protecting group (Fmoc)-grafted chitosan/Fmoc peptide hydrogel (FC/FI-Cur)	Dissolved into FC	Injection	*After 48 h,* ~68% *After 168 h,* ~82%	Female Sprague-Dawley Rats - Spinal Cord Transection	FC/FI-Cur accelerated DRG neurite outgrowth and SC migration in vitro. Modulation of inflammatory response, increased SC migration and remyelination in vivo
[121] *	Peptide hydrogel (HA-based with polypyrrole-coated fibers) (PM)-embedded human induced neural progenitor cells (iNPCs) with curcumin (PM-embedded iNPCs and CURC)	Mixed into PM	Local Placement over Spinal Cord	-	Female Sprague-Dawley Rats - Contusive Spinal Cord Injury Model	PPY-PM-iNPCs-CURC construct promotes neuron-like morphology in vitro and exhibits neuropreservation and decreases injured area in vivo
[63]	Curcumin-loaded polyacetal (PA)	Synthesized with PA	Intrathecal	pH 5.5 *After ~180 h*, ~100% pH 6.5 *After ~180 h,* ~50%	Sprague-Dawley Rats - Contusive Spinal Cord Injury Model	PA-curcumin increases neuroprotective effects and axonal growth, and promotes functional recovery with combined with epSPCs
[78] *	Curcumin-embedded matrix-metalloproteinase (MMP)-responsive triglycerol monostearate (TM) hydrogel	Embedded into poly(propylene sulfide)120	Endocranium Placement	* Dependent on MMP activity and ROS in vitro *After 14 days,* ~80% (CSF)	Albino ICR Mice - Weight Drop Traumatic Brain Injury Model	TM/PC reduced ROS and ROS-mediated effects, and brain edema, as well as exhibited anti-inflammatory effects; induced neuroregeneration

* Denotes a composite scaffold, where two or more biomaterial platforms are combined.

## Data Availability

Data sharing is not applicable to this review.
